# Validating k_Q_=1.0 assumption in TG51 with PTW 30013 farmer chamber for Varian TrueBeam's 2.5 MV imaging beam

**DOI:** 10.1002/acm2.12290

**Published:** 2018-03-01

**Authors:** Shelby Grzetic, Ahmet S. Ayan, Jeffrey Woollard, Nilendu Gupta

**Affiliations:** ^1^ Department of Radiation Oncology Ohio State University Columbus OH USA; ^2^ Advocate Christ Medical Center Oak Lawn IL USA

**Keywords:** 2.5 MV imaging dose, dose calibration

## Abstract

AAPM Report 142 recommends and the State of Ohio requires that the imaging dose be quantified in radiotherapy applications. Using the TG51 dose calibration protocol for MV Imaging dose measurement requires knowledge of the k_Q_ parameter for the beam quality and the ionization chamber type under investigation. The %dd(10)_x_ of the Varian TrueBeam 2.5 MV imaging beam falls outside the range of the available data for the calculation of the k_Q_ value. Due to the similarities of the 2.5 MV imaging beam and the ^60^Co beam, we and others made the assumption that k_Q_ = 1.0 in TG51 calculations. In this study, we used the TG21 and TG51 calibration protocols in conjunction to validate that k_Q_ = 1.0 for the 2.5 MV imaging beam using a PTW 30013 farmer chamber. Standard measurements for TG51 absolute dosimetry QA were performed at 100 cm SSD, 10 cm depth, 10 × 10 field size, delivering 100 Monitor Units to a waterproof Farmer Chamber (PTW TN30013) for both 2.5 and 6 MV. Both the TG21 and TG51 formalisms were used to calculate the dose to water per MU at d_max_ (D_w_/MU) for the 6 MV beam. The calculated outputs were 1.0005 and 1.0004 cGy/MU respectively. The TG21 formalism was then used to calculate (D_w_/MU) for the 2.5 MV imaging beam. This value was then used in the TG51 formalism to find k_Q_ for the 2.5 MV imaging beam. A k_Q_ value of 1.00 ± 0.01 was calculated for 2.5 MV using this method.

## 1 INTRODUCTION

AAPM Report 142^1^ recommends and the Ohio Department of Health (ODH) requires that all imaging dose be quantified for imaging when performed on patients during image guided radiation therapy (IGRT).^2^ The diagnostic (kV) beams have well‐established protocols to measure the doses to patients when they are used for imaging. Similarly, MV range treatment beams also have well‐established dose calculation protocols such as AAPM's Task Group‐51^3^ and the older Task Group‐21 protocols.^4^ These two protocols provide methodologies to calculate the dose to water for MV and ^60^Co beams. The absorbed‐dose‐to‐water factor, $N_{D,w}^{60Co}$, based on the TG51 protocol uses a k_Q_ factor which converts the calibration factor for a ^60^Co beam quality, for which the absorbed‐dose calibration factor is applicable, to a clinical beam quality of Q. In the TG51 protocol, k_Q_ values are provided in a figure and also tabulated for a variety of cylindrical chambers and beam qualities which are defined as the %dd(10)_x_. The later published Addendum to TG51^5^ provides an empirical formula to calculate the k_Q_ for clinical beams of quality with %dd(10)_x_ in the range of 63% to 86% and also provides k_Q_ values for some newer ionization chambers such as PTW TN30013 (PTW GmbH, Freiburg, Germany).

At our institution, we have Varian TrueBeam linear accelerators with 2.5 MV imaging beams. In an effort to meet the requirement of the ODH and to be able to characterize the dose given to patients during imaging with this beam, we set out to perform the dose quantification of the 2.5 MV imaging beam.

In order to have an accurate output measurement, k_Q_ must be known when using the TG51 formalism. We initially performed the calibration of this beam using the TG51 protocol with an assumed k_Q_ value of 1.0. A recently published paper by Gräfe et al.^6^ showed a similar calibration again with the assumed k_Q_ of 1.0, using the 2.5 MV imaging beam and 0.64 cc Exradin A12 (Standard Imaging Inc., Middleton, WI, USA) ionization chamber.

In order to validate our assumption of k_Q_ = 1.0 for the 2.5 MV imaging beam under consideration with the PTW TN30013 ionization chamber, we performed the calibration of the 2.5 MV with the older TG21 formalism, which does not require any knowledge of k_Q_. The aim of this study is to use the TG21 protocol for the absolute dosimetry calculation for the 2.5 MV beam to validate the assumed value for k_Q_ to be used in a TG51 protocol absolute dosimetry calibration.

Two previous studies have compared the doses calculated by the TG21 and TG51 protocols for megavoltage beam dosimetry. Cho et al.^7^ showed that for PTW N30001 & 23333 ion chambers, the TG51 to TG21 calculated dose ratio was 1.012 and 1.010 for ^60^Co and 6 MV photon beams respectively. Tailor et al.^8^ calculated the doses using both protocols for a variety of cylindrical chambers and photon beam energies. They showed that for the cylindrical chambers they tested the dose ratios were within ±1.0%, the highest being at the ^60^Co beam energy and decreasing with increasing photon energy.

## 2 MATERIALS AND METHODS

**Figure 1:**
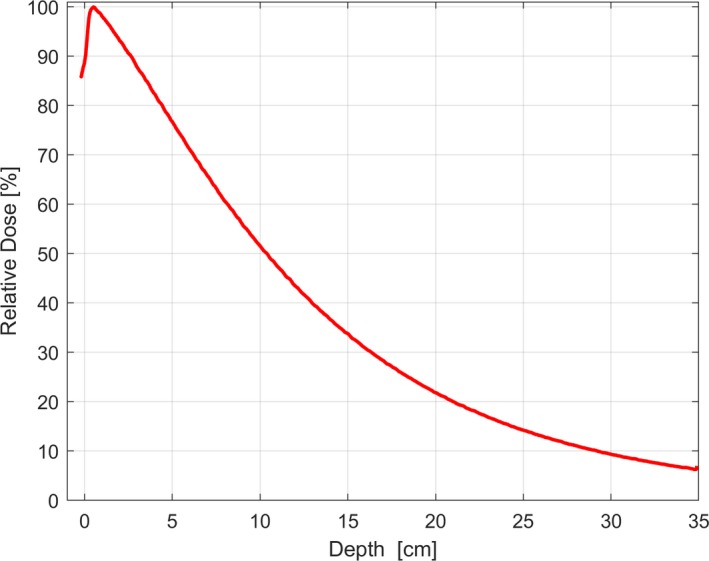
Measured PDD for the 2.5 MV imaging beam after shifting to the effective point of measurement.

We measured the percentage depth dose (PDD) of the 2.5 MV imaging beam of a Varian TrueBeam linear accelerator with a CC13 (IBA Dosimetry, Schwarzenbruck, Germany) detector in a cylindrical 3D Scanner water tank (Sun Nuclear Corporation, Melbourne, FL, USA) for a 10 × 10 cm field size at 100 cm SSD. Our measured %dd(10)_x_ for 2.5 MV is 51.53%. This is shown in Fig. 1. This value is outside the range of %dd(10)_x_ as shown in fig. 4 of the TG51 report or the empirical formula valid range as given in eq. (1) in the TG51 addendum. Measurements were then taken at 10 cm depth, 100 cm SSD, 10 × 10 cm^2^ field size with a PTW waterproof farmer chamber (TN30013) to calculate P_ion_. The exposure calibration factor, N_x_, and cavity‐gas factor, N_gas_, were taken from the ADCL calibration certificate of the ionization chamber used and were verified against a calculated value of N_gas_, using eq. (6) in TG21, assuming a PMMA (acrylic) wall and acrylic cap. P_wall_ was calculated using the mass stopping power ratio, L/ρ, and mean mass energy absorption coefficient, μ_en_/ρ, listed in the TG21 formalism for the wall material, acrylic, based on specifications from the manufacturer (74% PMMA, 26% graphite).^9^

In TG21 protocol, the dose to water is given by

(1) $$D_{W}=MN_{\mathrm{gas}}{\left(\overset{¯}{L}/\rho \right)}_{\mathrm{gas}}^{W}P_{\mathrm{ion}}P_{\mathrm{TP}}P_{\mathrm{elec}}P_{\mathrm{repl}}P_{\mathrm{wall}}$$

where

(2) $$P_{\mathrm{wall}}=\frac{\left[\alpha {\left(\overset{¯}{L}/\rho \right)}_{\mathrm{gas}}^{\mathrm{wall}}{\left(\overset{¯}{\mu }/\rho \right)}_{\mathrm{wall}}^{W}+\left(1-\alpha \right){\left(\overset{¯}{L}/\rho \right)}_{\mathrm{gas}}^{W}\right]}{{\left(\overset{¯}{L}/\rho \right)}_{\mathrm{gas}}^{\mathrm{med}}}$$

And

(3) $$N_{\mathrm{gas}}\left(Gy/R\right)=N_{x}\left(\frac{k\left(W/e\right)A_{\mathrm{ion}}A_{\mathrm{wall}}\beta_{\mathrm{wall}}}{\alpha {\left(L/\rho \right)}_{\mathrm{air}}^{\mathrm{wall}}{\left(\mu_{\mathrm{en}}/\rho \right)}_{\mathrm{wall}}^{\mathrm{air}}+\left(1-\alpha \right){\left(L/\rho \right)}_{\mathrm{air}}^{\mathrm{cap}}{\left(\mu_{\mathrm{en}}/\rho \right)}_{\mathrm{cap}}^{\mathrm{air}}}\right)$$

The fraction of ionization due to electrons from the chamber wall, α, was taken as zero using Fig. 1 of the TG21 protocol based on the nominal accelerating potential of 2.5 MV and the manufacturer‐specified chamber wall thickness of 0.056 g/cm^2^. P_repl_ was taken as 0.992 by using fig. 5 of the TG21 protocol. The factors and parameter values used in the TG21 calibration are listed in Tables 1 and 2. The first column of Tables 1 and 2 lists the corresponding item number in worksheet 1 and 2, respectively, in the TG21 protocol.

**Table 1:** Calculation of N_gas_ using both TG21 (Worksheet 1) and ADCL chamber calibration report

1.	Chamber Model	PTW TN30013
	Chamber wall thickness (g/cm^2^)	0.056
	Polarizing potential	+300V
2.	N_x_ (R/C)	5.64E+09
3.	k (C/kg R)	2.58E‐04
	W/e (J/C)	33.7
	β_wall_	1.005
4.	A_ion_	1.000
	A_wall_	0.990
	Α	0
	${\left(\overset{¯}{L}/\rho \right)}_{\mathrm{air}}^{\mathrm{wall}}$	1.103
	${\left(\overset{¯}{\mu }/\rho \right)}_{\mathrm{wall}}^{\mathrm{air}}$	0.928
	1‐α	1
	${\left(\overset{¯}{L}/\rho \right)}_{\mathrm{air}}^{\mathrm{cap}}$	1.103
	${\left(\overset{¯}{\mu }/\rho \right)}_{\mathrm{cap}}^{\mathrm{air}}$	0.925
5.	N_gas_ calculated from TG21 (Gy/C)	4.779E+07
	N_gas_ calculated from Calibration Certificate (Gy/C)	4.780E+07
	% Difference in N_gas_	0.01%

**Table 2:** Calculation of dose to water per MU at d_max_ (cGy/MU) according to TG21

1.	Nominal accelerating potential	2.5 MV	2.5 6 MV
2.	Phantom Material	Water	Water
	SSD	100 cm	100 cm
	Collimator Field Size (cm)	10 × 10	10 × 10
	Depth (cm)	10	10
3.2	Temperature (C)	22.5	22.5
	Pressure (mmHg)	739.3	739.3
	P_TP_	1.0297	1.0297
3.3	Raw Uncorrected Reading (C)	9.17E‐09	1.21E‐08
	Reading (C) corrected by P_TP_	9.44E‐11	1.24E‐10
3.4	Chamber Model	Farmer	Farmer
	Wall Material	PMMA + Graphite	PMMA + Graphite
	Inner Diameter (mm)	6.1	6.1
	N_gas_ (Gy/C)	4.78E + 07	4.78E + 07
3.5	${\left(\overset{¯}{L}/\rho \right)}_{\mathrm{air}}^{\mathrm{med}}$ (fig. 2, table IV)	1.135	1.127
3.6	P_wall_ (eq. 10)	1.002	1.000
	a (fig. 7)	0.45	0.25
	(1‐α)	0.55	0.75
	${\left(\overset{¯}{L}/\rho \right)}_{\mathrm{air}}^{\mathrm{wall}}$ (fig. 2, table IV)	1.0799	1.0706
	${\left(\overset{¯}{\mu_{\mathrm{en}}}/\rho \right)}_{\mathrm{air}}^{\mathrm{med}}$ (table IX)	1.111	1.11
	${\left(\overset{¯}{\mu_{\mathrm{en}}}/\rho \right)}_{\mathrm{air}}^{\mathrm{wall}}$ (table IX)	1.0522	1.052
	${\left(\overset{¯}{\mu_{\mathrm{en}}}/\rho \right)}_{\mathrm{wall}}^{\mathrm{med}}$	1.056	1.055
4.	P_ion_ (From TG51)	1.002	1.002
5.	P_Repl_ (fig. 5)	0.992	0.993
6.	D_med_/MU (eq. 9) – Gy/MU	0.0051	0.00667
7.2	ESC (table XIV)	1.000	1.000
7.3	${\left(\overset{¯}{\mu_{\mathrm{en}}}/\rho \right)}_{\mathrm{med}}^{\mathrm{water}}$ (table XII)	1.000	1.000
7.4	PDD at depth of measurement (%)	0.5153	0.6659
7.5	D_water_/MU (at d_max_) – Gy/MU (eq. 17)	0.009896	0.0100
	D_water_/MU (at d_max_) – cGy/MU	0.9896	1.0010

We calculated N_gas_ by using eq. (3) given above and also from

(4) $$N_{\mathrm{gas}}\left(Gy/R\right)=8.48*10^{-3}N_{x}A_{\mathrm{ion}}$$

which is provided on the ADCL calibration certificate and the manufacturer specification sheet [8]. The calculated values of N_gas_ are shown in Table 1.

After calculating (D_W_/MU)_TG21_ at the calibration dosimetry conditions using the TG21 protocol, we equated the calculated value to the TG51 equation used to calculate (D_W_/MU) for the same reference geometry and solved for k_Q_ as shown in eqs. (5) and (6):

(5) $$Reference\;dose\;from\;TG21=M_{\mathrm{raw}}P_{\mathrm{TP}}P_{\mathrm{ion}}P_{\mathrm{elec}}P_{\mathrm{pol}}k_{Q}N_{D,W}^{Co^{60}}$$

(6) $$k_{Q}=\frac{{}_{Reference\;dose\;from\;TG21\left[\frac{\mathrm{cGy}}{\mathrm{MU}}\right]}}{M_{\mathrm{raw}}P_{\mathrm{TP}}P_{\mathrm{ion}}P_{\mathrm{elec}}P_{\mathrm{pol}}N_{D,W}^{Co^{60}}}$$

Numeral values for this calculation are shown in Table 3. As a validation of the method, the same process was applied for the 6 MV beam.

**Table 3:** Calculation of dose to water per MU at d_max_ according to TG51 in addition to derivation of TG21 calculated k_Q_ value

Measurements from TG51	2.5 MV	6 MV
M_raw_ (C)	9.17E‐09	1.21E‐08
P_TP_	1.0297	1.0297
P_ION_	1.002	1.00222
P_elec_	1.002	1.002
P_pol_	1.000	0.99959
N_D,W_^60Co^	5.38E + 07	5.38E + 07
Clinical %dd(10)_x_	0.5153	0.6659
MU delivered	100	100
TG21 calculated k_Q_	1.0002	0.993
TG51 addendum – calculated k_Q_	1.000	0.9919
% Difference	0.02%	0.06%

## 3 RESULTS

We calculated the absorbed dose ratio at the reference conditions as (TG51/TG21)_Dose_ = 0.9994 for the 6 MV beam using the PTW 300013 ion chamber. Tailor et al.^8^ showed that (TG51/TG21)_Dose_ = 1.003 for a 6 MV beam using PTW N30006 ion chamber. The N30006 is equivalent to PTW 30013 according to the manufacturer's specifications.^9^ Our result differs from Tailor et al.'s prediction by only 0.3%. Hence, we hypothesize that our PTW N30013 chamber material dependent TG21 protocol parameters (L/ρ) and (μ_en_/ρ) are accurate.

Next, by calculating the absorbed dose of the 2.5 MV imaging beam with the TG21 formalism and solving eq. (6), k_Q_ value was calculated as 1.0002 (Table 3).

## 4 CONCLUSION

The method outlined yielded a k_Q_ value of 1.0002 for the 2.5 MV TrueBeam imaging photon beam using the PTW TN30013 ionization chamber. This value is within 0.02% of our and Gräfe et al.'s assumed k_Q_ = 1.0. With up to ±1% difference shown^8^ between the dose calibration for photon beams by using TG21 and TG51 protocols for ^60^Co to 18 MV photon energies, we assigned a 1% uncertainty in our calculation of k_Q_. The use of a k_Q_ = 1.000 is adequate for the 2.5 MV imaging photon beam using the PTW TN30013 ionization chamber to characterize the imaging beam dose.

## CONFLICT OF INTEREST

The authors have no conflicts of interest relevant to the content of this article.
